# Electroconvulsive Therapy Induces Neurogenesis in Frontal Rat Brain Areas

**DOI:** 10.1371/journal.pone.0069869

**Published:** 2013-07-26

**Authors:** Dragos Inta, Juan M. Lima-Ojeda, Thorsten Lau, Wannan Tang, Christof Dormann, Rolf Sprengel, Patrick Schloss, Alexander Sartorius, Andreas Meyer-Lindenberg, Peter Gass

**Affiliations:** 1 Department of Psychiatry and Psychotherapy, Central Institute of Mental Health, Medical Faculty Mannheim, University of Heidelberg, Mannheim, Germany; 2 Biochemical Laboratory, Central Institute of Mental Health, Medical Faculty Mannheim, University of Heidelberg, Mannheim, Germany; 3 Department of Molecular Neurobiology, Max-Planck-Institute of Medical Research, Heidelberg, Germany; Max Planck Institute of Psychiatry, Germany

## Abstract

Electroconvulsive therapy (ECT) is an effective therapy for several psychiatric disorders, including severe major depression, mania and certain forms of schizophrenia. It had been proposed that ECT acts by modulating local plasticity via the stimulation of neurogenesis. In fact, among antidepressant therapies, ECT is the most robust enhancer of neurogenesis in the hippocampus of rodents and non-human primates. The existence of ECT-triggered neurogenesis in other brain areas, particularly in those adjacent to the other main locus of neurogenesis, the subventricular zone (SVZ), had so far remained unknown. Here we show that ECT also strongly enhances neurogenesis in frontal brain areas, especially in the rostro-medial striatum, generating specific, small-size calretinin-positive interneurons. We provide here the first evidence that ECT stimulates neurogenesis in areas outside the hippocampus. Our data may open research possibilities that focus on the plastic changes induced by ECT in frontal limbic circuitry.

## Introduction

ECT is the therapy of choice for treatment-refractory depression, as well as for treatment of acute mania and catatonic schizophrenia [1]. The mechanisms underlying the therapeutic effect of ECT are not yet understood. Because antidepressant treatment attenuates stress-induced structural alterations via signal transduction pathways linked to neuronal plasticity, it has been proposed that synaptic plasticity plays a prominent role in the pathophysiology of depression [2]. An important hypothesis of the neurobiological origins of depression suggests that neurogenesis in the hippocampal formation may represent an important factor, in the precipitation of, and recovery from, episodes of clinical depression [3]. Supporting this view, ECT robustly stimulates neurogenesis in the hippocampus of rodents, much stronger than other antidepressant therapies [4]. In terms of the potential clinical relevance of these changes, the potent stimulatory effect of ECT on hippocampal neurogenesis has also demonstrated in non-human primates [5].

The occurrence of neurogenesis in other brain areas following ECT is unknown. The anterior rodent SVZ is, in addition to the hippocampal dentate gyrus, a primary site of adult neurogenesis [6], generating throughout life GABAergic interneurons that migrate along the rostral migratory stream to the olfactory bulb. Recent studies have shown that the early postnatal SVZ is also a reservoir for small size calretinin-positive interneurons in cortical and subcortical brain areas located adjacent to the SVZ, morphologically similar to newborn interneurons migrating to the olfactory bulb [7]. Calretinin (CR) is a calcium-binding protein expressed in several subpopulations of GABAergic interneurons [8]. In the olfactory bulb newborn granule cells expressing high levels of CR represent the vast majority of newly-generated neurons in the postnatal and adult SVZ [9]. Therefore, it is not surprising that the early postnatal SVZ generates predominantly this interneuronal subtype in adjacent cortical and subcortical regions as well. Importantly, the process of neurogenesis/migration outside the SVZ/RMS/OB axis is not limited to early postnatal stages. Newborn interneurons with similar neurochemical and morphological features have been detected, although in a considerably lower number, in cortical and/or subcortical regions adjacent to the SVZ in adult rats and rabbits [10], [11]. Moreover, also in the adult, different triggers, such as ischemia and brain trauma, robustly stimulate neurogenesis in the SVZ [12], [13], generating exclusively CR-positive interneurons in regions around the SVZ/RMS, especially in the striatum. Considering these results and the fact that stimulation of SVZ neurogenesis may induce plastic changes in adjacent limbic regions implicated in mood regulation, we investigated, using bromodeoxyuridine (BrdU) birthdating, whether ECT induces neurogenesis in extra-hippocampal regions. We used a classical paradigm of chronic ECT in rodents [14], as repeated administration of electroconvulsive seizures is necessary for its clinical efficacy, a fact suggesting that long-term changes at the network level are required for the therapeutic effect.

## Materials and Methods

### ECT Procedure

Three-month old male Sprague-Dawley rats (Janvier, France) were housed under standard conditions and received ECT (once daily for 10 day) (100 mA, 50 Hz, for 1 s with pulse width 500 ms) via earclip electrodes after pretreatment with an electrode gel, as described previously [14], [15]. To assess the effect of a single electroconvulsive seizure, one cohort of rats was treated with single ECT exposure. Rats were monitored after treatment to ensure that it consistently resulted in tonic-clonic seizures lasting a minimum of 20 s. Sham-treated animals were handled similarly, had earclips mounted but no current was passed. All animals (n = 6 per group) were sacrificed (S) and perfused 10 days, 28 days or 42 days following ECT treatment. All experiments had been approved by the German Committee on Animal Care and Use, according to the European Communities Council Directive of 24 November 1986 (86/609/EEC).

### Birth-Dating Analysis

**Figure 1:**
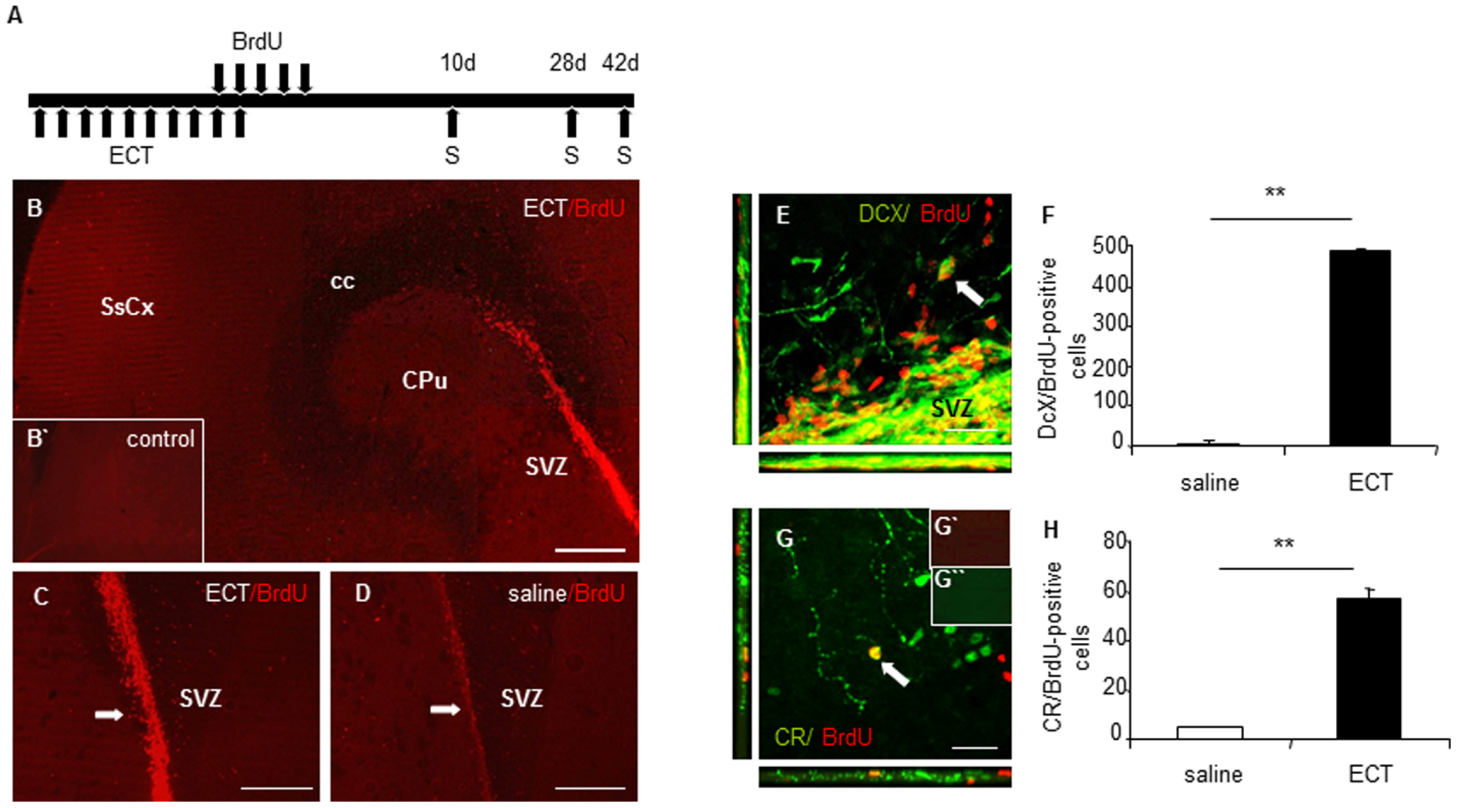
ECT significantly increases BrdU expression in the SVZ and the number of newborn DCX-positive neuroblasts and CR-positive interneurons in the rat striatum and nucleus accumbens. (A) Schematic representation of the ECT and BrdU applications and the analysis of animals (sacrification, S) 10d (migration) or 42d (differentiation) after the last BrdU injection (B–D) A significant increase in BrdU expression, indicative of intense cell proliferation, 10 days after ECT in the SVZ (arrows) (B, C) compared to saline-treated animals (D). BrdU expression was prominent in the SVZ and proximal areas and low in other areas (B). As expected, no specific staining was visible in control sections processed only with the secondary antibody (B`). (E,F) Significant increase in the number of DCX/BrdU-positive cells (green/red) in the medial striatum in ECT-treated animals compared to saline-treated rats 10d after the BrdU treatment, quantitative analysis in (F). (G) Example of a double-labelled CR/BrdU interneuron in the nucleus accumbens (*arrow*) 42d after the BrdU treatment. (G`, G`) No specific staining was visible in control sections processed only with the secondary antibodies. (H) Quantitative analysis of the CR/BrdU co-expression in ECT-treated vs. saline-treated animals. Statistical differences determined by Mann-Whitney U test (0.001<**p<0.01). Scale bars: B–D, 200 µm, E, G, 20 µm. SsCx, somatosensory cortex; cc, corpus callosum; CPu, caudate-putamen; SVZ, subventricular zone.

Rats receiving 10 electroconvulsive seizures were injected intraperitoneally (i.p.) with 100 mg/kg BrdU twice per day (at 9∶00 a.m. and 5 p.m.) for 5 days beginning on Day 8 of the ECT treatment and continuing 3 days following the last ECT (see Figure 1A). Animals treated with one electroconvulsive seizure similarly received 100 mg/kg BrdU twice per day for 5 days, starting 2 h after the seizure. Animals were sacrificed at different time points: after 10 days (for the cohort treated with one seizure) and 10 days, 28 days or 42 days after the last BrdU injection for the ECT cohorts that received 10 seizures. Animals were sacrificed by transcardiac perfusion with 4% paraformaldehyde and brains were post-fixed overnight [16].

### Immunohistochemistry

To evaluate intermediate stages of neurogenesis, we used an established marker for identifying newborn migrating neurons, doublecortin (DCX) [17]. For this purpose, 50 µm coronal free floating vibratome sections were processed as described previously [18] and washed in 0.1 M phosphate buffer saline containing 0.3% Triton X-100 (PBST), pH 7.4, immersed in 0.3% hydrogen peroxidase for 20 min, and washed again before being incubated for 24 h at 4°C with the respective primary antibody: rat anti-BrdU, 1∶400 (Accurate), goat anti-DCX, 1∶400 (Santa Cruz) or mouse anti-CR antibody, 1∶5,000 (Swant), alone or in combination. We used as secondary antibodies Alexa 488- or Alexa 555-conjugated anti-mouse, anti-goat or anti-rat secondary antibodies, respectively (Invitrogen). As technical controls, separate sections in each staining were labelled with secondary antibodies only.

### Cell Counting

Labelled cells were quantified from coronal sections in the medial rostral striatum. Every sixth section from coordinates 3.70 to 1.70 mm relative to Bregma was quantified, representing four sections per brain. Cell counting was performed by an investigator blind to the treatment using a LEICA confocal laser scanning microscope (LEICA TCS-NT), similar to the procedure described previously [12]. The demonstration of the occurrence of neurogenesis via identification of co-labeling by BrdU and neuronal markers is difficult, particularly in small-size CR-positive cells generated in the postnatal/adult cortex [7], [10],[12],[19]. Satellite glial cells can lie in the immediate vicinity of these small neurons. Therefore, the demonstration of co-labeling requires thorough examination of individual neurons by an experienced investigator using confocal microscopy, scanning systematically the entire cell to examine Z-projections. Confocal Z sectioning was performed using 40× oil-immersion and 63× oil-immersion objectives. All secondary antibody combinations were carefully examined to ensure that there was no cross-reactivity between them. Images from a Z-stack were 3-D reconstructed using the Leica TCS software. To demonstrate co-expression, each figure depicts BrdU co-localization with DCX or CR and full thickness xy-projections of the Z-stack are shown. Overview images of immunhistochemistry-labeled sections with lower magnification (10×) were acquired using a Zeiss Axioskop 2 plus microscope.

### Statistical Analysis

Statistical analysis of changes in the number of BrdU/DCX, BrdU/CR, DCX or CR-positive cells after ECT treatment was performed after testing the normality of the distribution of the data, using the Mann-Whitney U test, and the statistical significance level was determined at *P*<0.05. Data are expressed as the mean±S.E.M.

## Results

### ECT Induces a Robust Increase in the Number of Newborn Neuroblasts in the Striatum Adjacent to the SVZ

ECT administered to adult rats for 10 days (Figure 1A) evoked a strong increase in the number of BrdU-positive cells in the SVZ as compared to saline-treated animals 10 days after the last BrdU injection (Figure 1B–D). Many BrdU-positive cells co-expressing the marker for migrating neuroblasts DCX were located in the SVZ and in cells apparently detaching into adjacent striatal areas (Figure 1E). We identified much more DCX/BrdU-positive neuroblasts in the medial striatum of ECT-treated rats, compared to very few DCX/BrdU-expressing cells in sham-treated animals (Figure 1F). A quantitative analysis revealed a robust increase in the number of DCX/BrdU-positive cells in ECT-treated animals, as compared to sham-treated rats (485±8.83 *vs.* 10.17±1.08; p = 0.002). A few scattered BrdU/DCX-positive cells were detected in the lateral septum and nucleus accumbens, but not in adjacent cortical areas. In rats treated with a single electroconvulsive seizure, the increase in the number of DCX/BrdU-positive cells after 10 days was significantly higher in ECT-treated rats than in sham-treated animals (78,5±2.49 *vs.* 11.5±1.38; p = 0.002), but clearly less than the level obtained after the application of 10 electroconvulsive seizures.

### Numerous Newborn CR-positive Interneurons in the Medial Striatum post-ECT

To analyze the differentiation of newborn neurons generated by ECT, we investigated rats at 4 weeks and 6 weeks after ECT for co-expression of CR and BrdU. Most CR/BrdU-positive interneurons were located in the medial striatum (data not shown). Few CR/BrdU cells with similar morphological characteristics (small, rounded soma, with 1–2 processes) were also present in the nucleus accumbens and lateral septum following ECT (Figure 1G). A quantitative analysis of CR/BrdU-co-expression was performed in the medial striatum and demonstrated robustly increased neurogenesis and differentiation into specific interneurons at similar levels between ECT-treated *vs.* sham-treated animals both at 4 (67.33±2.22 *vs.* 6.5±0.76; p = 0.002) and 6 weeks post-ECT (57.17±3.15 *vs*. 4.33±0.92; p = 0.002, Figure 1H).

### General Increase in the Number of Striatal Neuroblasts and Accumulation of CR-positive Interneurons in the Striatum Induced by ECT

**Figure 2:**
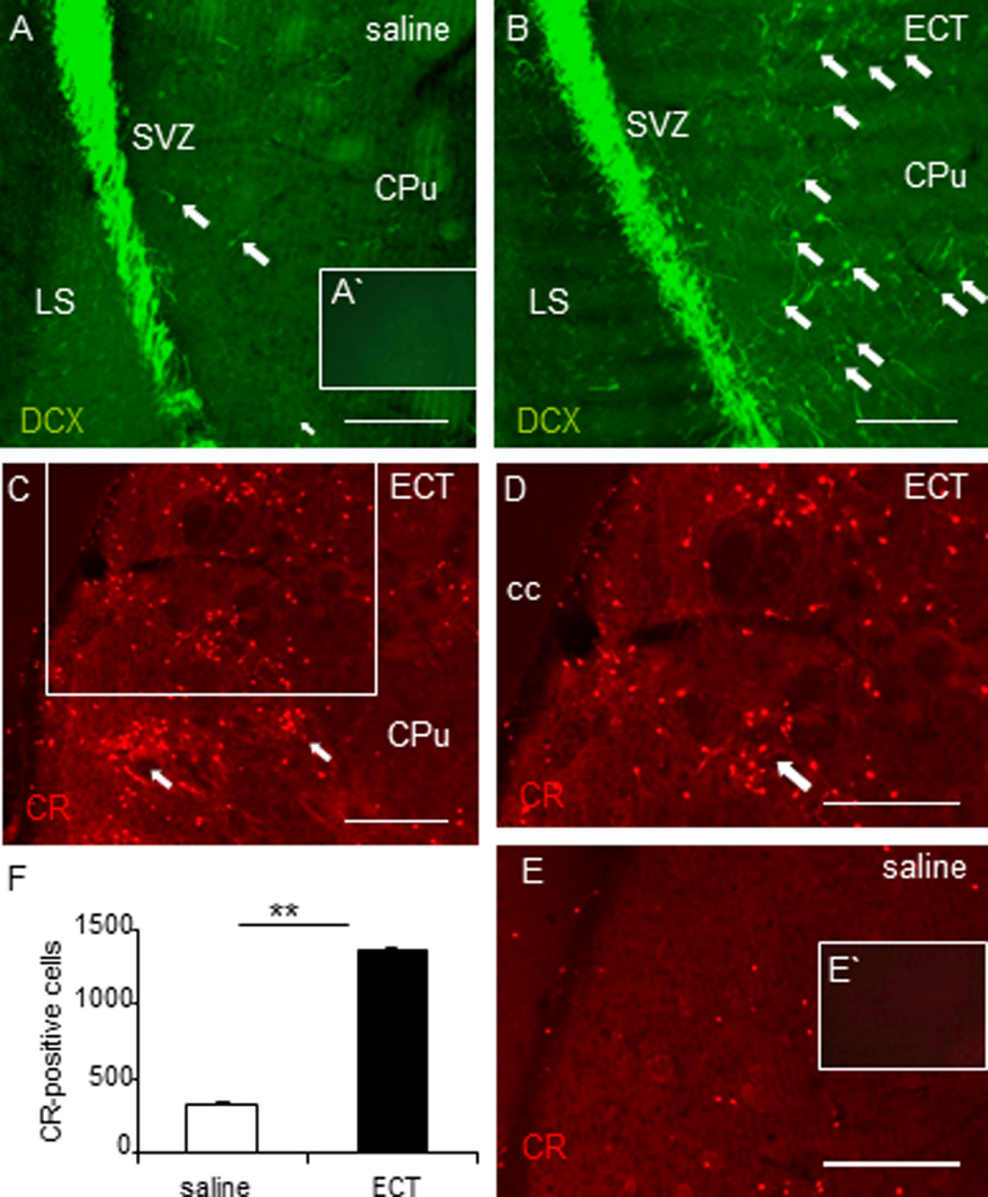
ECT increases the total number of both DCX-positive neuroblasts and CR-positive interneurons in the rat striatum. (A,B) Significant increase in the number of DCX-positive cells (green) in the medial striatum (*arrows*) in comparison to controls (A) in ECT-treated animals (B) 10d after the BrdU treatment. (A) No specific staining was visible in control sections processed only with the secondary antibody. (C–E) ECT augments the number of CR-positive interneurons (red) often grouped in clusters (*arrows*) in the dorso-medial striatum (C, D is an insight of C) compared to controls (E). (E) No specific staining was visible in control sections processed only with the secondary antibody. (F) Quantitative analysis of the CR expression in ECT-treated vs. control animals. Statistical differences determined by Mann-Whitney U test (0.001<**p<0.01). Scale bars: 200 µm. CPu, caudate-putamen; LS, lateral septum; SVZ, subventricular zone.

Due to its short half-life time of a few hours, BrdU incorporation may reflect only a glimps of the stimulatory effect of ECT on neurogenesis. Therefore, we also determined whether ECT increased the total number of neuroblasts and CR-positive interneurons in the medial striatum. We found that, similar to neuroblasts incorporating BrdU, ECT-treated rats demonstrated a strong increase in the total number of DCX-positive cells (Figure 2A,B). Similarly, ECT-treated rats displayed significantly more striatal CR-positive interneurons (Figure 2C,D) than sham-treated animals (Fig. 2E). Interestingly, CR-positive interneurons were often grouped in clusters in the medial and dorsal striatum, especially at the level of the rostral pole of the corpus callosum (Figure 2C–E, quantitative analysis of CR-expression in ECT-treated animals compared to controls is provided in Figure 2F).

## Discussion

The present results demonstrate for the first time the occurrence of an unexpected and robust ECT-induced neurogenesis outside the hippocampus, opening new avenues for research focusing on plastic changes induced by ECT in limbic areas. Previously, enhanced gliogenesis and proliferation of endothelial cells, following ECT, but no neurogenesis, had been reported in the frontal cortex, suggesting a lack of effect of ECT on SVZ neurogenesis [20]. Another study analyzing the effect of pilocarpine-induced status epilepticus on SVZ neurogenesis, reported increased cell proliferation and ectopic migration of neuroblasts into adjacent regions [21]. These results differ, however, significantly from the present data, since Parent and colleagues, despite finding a significant number of neuroblasts migrating ectopically from the SVZ 1–2 weeks after chemoconvulsant-induced seizures, reported that most newly-generated neurons disappeared 35 days later [21]. In contrast, we found a high number of newborn neurons at 10 days, 28 days and 42 days post-ECT. These discrepancies could result from the minor protocol differences, the region analyzed and/or from the markers used to identify newborn neurons (CR vs. NeuN). Parent et al. used a lower dose of BrdU than in our study (50 mg/kg vs. 100 mg/kg) and administrated it either twice per day on days 1, 4, 7, 14, 35 following pilocarpine treatment, or in a second protocol by three injections over 6 hours on day 7 following pilocarpine treatment. In our protocol, BrdU was administered twice per day on 5 consecutive days, partly overlapping with the ECT (Figure 1A). Our protocol may better cover the period of high neurogenesis immediately after the ECT-induced seizures. In addition, striatal CR-positive interneurons, generated at low level under physiological conditions, express very low or non-detectable levels of NeuN [22]; therefore analysis relying only on this neuronal marker, together with omitting intermediate stages of neurogenesis (e.g. neuronal migration) may fail to detect neurogenesis. We also cannot exclude the possibility that chemoconvulsant-triggered seizures does not result in the same level of SVZ neurogenesis as ECT. Additionally, we found a significant reduction in the number of newborn neurons from migration to differentiation (CR-positive interneurons compared to DCX-positive neuroblasts) (see also [10]), possibly leading in a situation with low cell proliferation to detection of migrating neuroblasts, but not of consecutively differentiating neurons. Furthermore, one electroconvulsive seizure induced a significantly lower number of newborn neurons than a series of 10 repeated seizures. Regarding the dynamics of the process described here, we found that many striatal CR-positive interneurons generated following ECT were grouped in clusters. It would be interesting to determine the mechanisms underlying the migration of newborn neurons and their clustering. In the early postnatal brain, blood vessels serve as scaffolding for the migration of SVZ-derived immature neurons [23]. The existence of clusters of CR-positive neurons raises questions about the potential relationship between these tightly-associated neurons. Le Magueresse and colleagues showed that CR-positive interneurons generated in the early postnatal SVZ are integrated into local circuits [24]. The function of newly-generated neurons post-ECT, however, remains to be established. In addition, ECT influences not only neurogenesis, but also angiogenesis [25], gliogenesis and glial cell activation [26], [27] and recruitment of blood-derived macrophages [28]. Further investigation of the potential role of these processes in modulating the function and connectivity of newly-formed neurons may lead to a better understanding of the neurobiological changes triggered by ECT.

Our findings could be of importance regarding plastic changes induced by ECT in frontal limbic circuitry, especially in areas adjacent to the SVZ, such as perigenual regions. The medial striatum is part of the limbic system, with afferents from the medial prefrontal and cingulate cortices [29]. Despite neuroanatomical differences, our data may also be relevant for primates. Hyperactivity of the subgenual cingulate cortex occurs in depression, and antidepressant therapies such as ECT and deep brain stimulation (DBS) of the subgenual cortex, reverse this hyperstimulation [30], [31], [32]. Interestingly, it has been proposed that DBS may act by increasing GABAergic inhibition via CR-positive interneurons [31]. Interestingly, the proportion of this type of interneuron is increased considerably in humans as compared to rodents [33]. However, these interneurons have been much less studied than other interneuronal subtypes in the striatum [34]. Future research will focus on an understanding of the function of these interneuronal subtypes, and their impact on local circuits and their role in the therapeutic effect of ECT.
